# Chemistry and Charge Trapping at the Interface of Silver and Ultrathin Layers of Zinc Oxide

**DOI:** 10.1021/acsami.1c11566

**Published:** 2021-10-09

**Authors:** M. Rahamim, H. Cohen, E. Edri

**Affiliations:** † Department of Chemical Engineering, Ben-Gurion University of the Negev, Be’er-Sheva 8410501, Israel; ‡ Department of Chemical Research Support, Weizmann Institute of Science, Rehovot 7610000, Israel; § Ilse Katz Institute for Nanoscale Science and Technology, Be’er-Sheva 8410501, Israel; ∥ Blechner Center for Industrial Catalysis and Process Development, Be’er-Sheva 8410501, Israel

**Keywords:** Ag−ZnO, thin films, interface defect states, atomic layer deposition, surface photovoltage

## Abstract

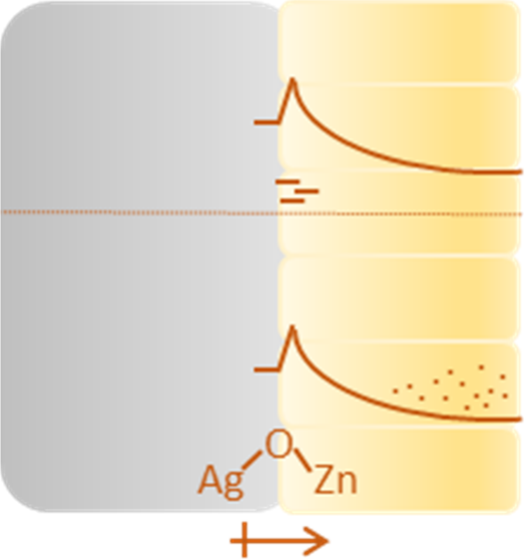

Zinc oxide, a wide-band-gap semiconductor, shows intriguing optoelectronic properties when coupled with Ag. Specifically, an absorbance band in the visible range that is not apparent in the separated materials emerges when the interface is formed. Interestingly, photoexcitation of this “interface band” or band-to-band results in a counterintuitive photovoltaic response when a supra/sub-band-gap light is shone. To investigate the origin of this absorbance band and photovoltaic response, we studied in detail the energy-band alignment of ultrathin layers of ZnO (3–60 nm) with Ag. Our analysis indicated that an ‘electrostatic potential cliff’ is formed within the first 1–2 nm of ZnO. In addition, oxygen vacancies, presumably generated by Ag*_x_*O–Zn bonds, form mid-gap acceptor states within these first few nm. Both effects facilitate a valence band-to-defect state optical transition that is confined to the interface region. The second type of defects—hole-trap states associated with zinc hydroxide—are spread throughout the ZnO layer and dominate the supra-band-gap photovoltaic response. These findings have potential implications in emerging technologies such as photocatalytic Ag/ZnO heterostructures that will utilize the long-lived charges for chemical work or other optoelectronic applications.

## Introduction

Metal/metal-oxide interfaces are prevalent in modern devices such as diodes, sensors, photovoltaic devices, and emerging technologies such as photocatalysis and metal-oxide electronics.^1−13^ These interfaces often cause beneficial or deleterious effects, such as hot electron transfer or charge trapping at interface defects.^14−16^ Zinc oxide has superior electronic qualities, and it is used in numerous applications.^17,18^ The interface of Ag and ZnO shows intriguing optoelectronic properties^9,19−21^ but employing them requires better understanding of the interface structure and function.

Surface plasmon resonance (SPR)^22−24^ and surface plasmon polariton (SPP)^25−27^ coupled with electron transfer^28,29^ and trapping at interface defect states^30−33^ take place at the Ag/ZnO interface. SPPs enhance the local electric fields, have a characteristic optical absorbance band,^9,20^ and were employed to enhance the transmittance of thin multilayer stacks.^34^ In another study, SPPs increased the charge separation efficiency and surface photovoltage (SPV) in Ag/ZnO gratings.^27^ Alternatively, SPR increases the electron density at the ZnO defect states and enhances visible-light emission.^22^ Brillson and co-workers,^35,36^ and Durbin and co-workers,^37^ have studied and emphasized the electronic implications of defect states at metal/ZnO interfaces. Silver has a slightly positive heat of interface reaction and is not considered a reactive metal. Therefore, ZnO/Ag interfaces appear to have an interface index close to one.^36,38,39^ Yet, ultrathin layers of Ag*_x_*O can form near the interface as a metal oxide upon reaction with lattice oxygen, which would lead to oxygen vacancies in the metal oxide, and hence mid-gap states and energy-level pinning.

At room temperature, ZnO has a hexagonal wurtzite-type crystal structure. The alternating Zn^2+^ and O^2–^ layers along the *c*-axis in the hexagonal wurtzite structure induce a prominent polar character in the (001) direction.^40^ The native polarity of the crystal can guide the crystal growth direction and the structure of the interface that forms with other materials. Additionally, when a metal is deposited on a single crystal (e.g., by sputtering), the interface can undergo reactions and deformation due to heating or reactive species, which can cause defects that are specific to the deposition method. Comparing the properties of interfaces created by various deposition techniques is beneficial to understanding the interface structure and function. Several techniques were used for depositing thin films of ZnO^17,18,41,42^ and Ag/ZnO nano-heterostructures.^9,24,29,43,44^ Nevertheless, unless particular care is taken, the interface of Ag/ZnO nanostructures does not have a specific orientation, obscuring the effect of the native crystal polarity. Also, due to the high surface-to-volume ratio in nano-heterostructures, the consequences of surface defects can mask other effects resulting from a “bulk”, or specifically the interface. In other studies of the Ag/thin-film ZnO interface, the ZnO films were thicker than 100 nm, discontinuous, or lacked any preferred orientation,^36,45^ which limited the study of interface properties. Atomic layer deposition (ALD) is a popular thin-film deposition technique. The sequential and self-limiting surface reactions result in a conformal deposition with sub-nanometer control of the film thickness,^46,47^ enabling the study of Ag/ZnO interfaces with precisely tunable film thickness that helps to separate interface from bulk effects. The ALD reaction conditions and the process parameters are influential factors in the film properties. For example, the preferred orientation of the grains in the ZnO film is affected chiefly by the deposition temperature, the precursors’ type, and the purge time length.^48−51^ Furthermore, the ZnO properties and stoichiometry can be tuned by the deposition temperature or the use of plasma during deposition.^52−54^

Surface photovoltage is a contactless method based on the Kelvin probe technique. It is used for measuring the contact potential difference (CPD) between a surface and a probe. Changes in the contact potential difference with respect to the ground state, for example, by photoexcitation, indicate accumulation of charge at the surface. The sign of the change (positive or negative) in the contact potential difference indicates the type of charge effectively accumulated at the surface i.e., positive or negative charge.^55,56^ In this study, we ALD-deposited 3–60 nm thick continuous ZnO films with a distinguished preferred orientation of the *c*-axis perpendicular to the substrate surface. By comparing the composition, structure, and optoelectronic response of the Ag/ZnO systems with several ZnO thicknesses, we differentiate between effects that stem directly from the Ag/ZnO interface and those that stem from the bulk of ZnO. This approach enabled us to shed light on the interfacial structural and chemical properties, with implications on the optical and electronic properties of the combined system.

## Experimental Section

### Thin-Film Deposition

Glass substrates of dimensions 9.0 × 26.0 mm^2^ were cleaned by a piranha solution before coating, sequentially, with 10 nm of Ti and 100 nm of Ag by E-gun evaporation (VST, TFDS-462B). Thin ZnO films with different thicknesses were deposited on top of the Ag layer by atomic layer deposition (ALD; ARRADIANCE, GEMStar XT). The deposition temperature was 125°C with 10 SCCM of Ar as the carrier gas; dimethylzinc (DMZ; STREM, 99%) and water (ThermoFisher Scientific Barnstead MicroPure, 18.2 MΩ·cm) were used as the precursors. The pressure in the deposition chamber was ∼180 mTorr, while the peak pressure in a single DMZ pulse was ∼680 mTorr and that in a single water-vapor pulse was ∼760 mTorr. A single cycle deposition consists of a 21 ms pulse of DMZ, 2.5 s of closing the expo valve, 21 s of purge, 80 ms pulse of water, 2.5 s of closing the expo valve, and 80 s of purge. In all, 50, 100, and 300 cycles corresponding to 10 nm, 20 nm, and 60 nm of ZnO, respectively, were deposited at a growth rate of 0.20 ± 0.05 nm/cycle.

### Characterization

Diffused reflectance using an integrating sphere and transmission were measured in the range of 200–800 nm using a Cary 5000 spectrophotometer (Agilent). Ag deposited on top of a clean glass substrate was used in the parallel (blank) beam position for baseline correction for obtaining the Ag/ZnO samples’ diffused reflectance spectrum. For transmission measurements, the parallel beam position was empty. Raman and photoluminescence (PL) measurements were done with a Horiba LabRam HR evolution micro-Raman system, equipped with a Synapse Open Electrode CCD detector. The excitation source for the PL was a 325 nm laser, and for the Raman spectroscopy, 325 and 532 nm lasers were used. The 532 nm laser, with a power on the sample of 3 mW, was focused using an 100× objective onto a spot of about 1.3 μm, and the spectra were collected using a 100 μm confocal microscope hole with a grating of 1800 g/mm. The typical exposure time was 120 s. The 325 nm laser with a power on the sample of 0.6 mW was focused using an 40× objective onto a spot of about 1 μm, and the spectra were collected using a 200 μm confocal microscope hole with a grating of 1800 g/mm. The typical exposure time was 60 and 0.5 s for the Raman and the PL measurements, respectively. The X-ray diffraction (XRD) patterns of the samples were recorded using PANalytical’s Empyrean multipurpose diffractometer, with Cu K_α1_ (λ = 1.54 Å) radiation wavelength at a scanning rate of 7°/min for 2θ ranging from 10° to 90°. The Ag/ZnO interface was investigated using the FEI Talos F200C transmission electron microscope (TEM) operating at 200 kV. The images were taken with an FEI Ceta 16M CMOS camera. The samples’ fabrication for the TEM measurements was carried out using a Helios G4 UC focused ion beam (dual-beam FIB) tool.

X-ray photoelectron spectroscopy (XPS) measurements were performed using a Kratos AXIS-Ultra DLD spectrometer, with a monochromatic Al kα source at 15–75 W and detection pass energies of 20–80 eV. The base pressure in the analysis chamber was 5 × 10^–10^ torr. The work-function values were extracted from the spectral onset of the secondary electron emission (SEE) spectrum. Beam-induced charging issues were addressed by several complementary means. First, a conductive carbon tape was used to get electrical contact to the top surface. Second, measurements at varied charging conditions (varied X-ray source power, as well as the application of an electron flood gun, eFG) were performed, so that line shifts of the overlayer elements (Zn, O, C) could be compared with those of the metallic silver (Ag).^57,58^ Third, by measuring the work function (WF) of the sample under an extremely low X-ray flux (source power of 0.3 W and bias voltage of −10 V), and by repeating these measurements a number of times during every experiment, we could identify the charge-free state of the sample quite accurately.^59^ Finally, the evaluation of irreversible beam-induced effects (e.g., sample damage and electrostatic effects) was done by comparing the measurements on fresh spots with multiple repeated scans on a given spot. Unless specified otherwise, all photoelectron measurements were taken with a normal (0°) emission angle. For the 3 nm thick ZnO layer, measurements at an emission angle of 60° were performed as well. Ar^+^ ion sputtering was applied for the bare Ag surface so as to remove the native contaminants and get a decent reference of the substrate.

Contact potential difference (CPD) measurements were performed using the noncontact Kelvin probe configuration (KP Technology Ltd.). Light-emitting diodes (LED; 370 and 450 nm) were used as the photon source for surface photovoltage measurements.

## Results

**Figure 1:**
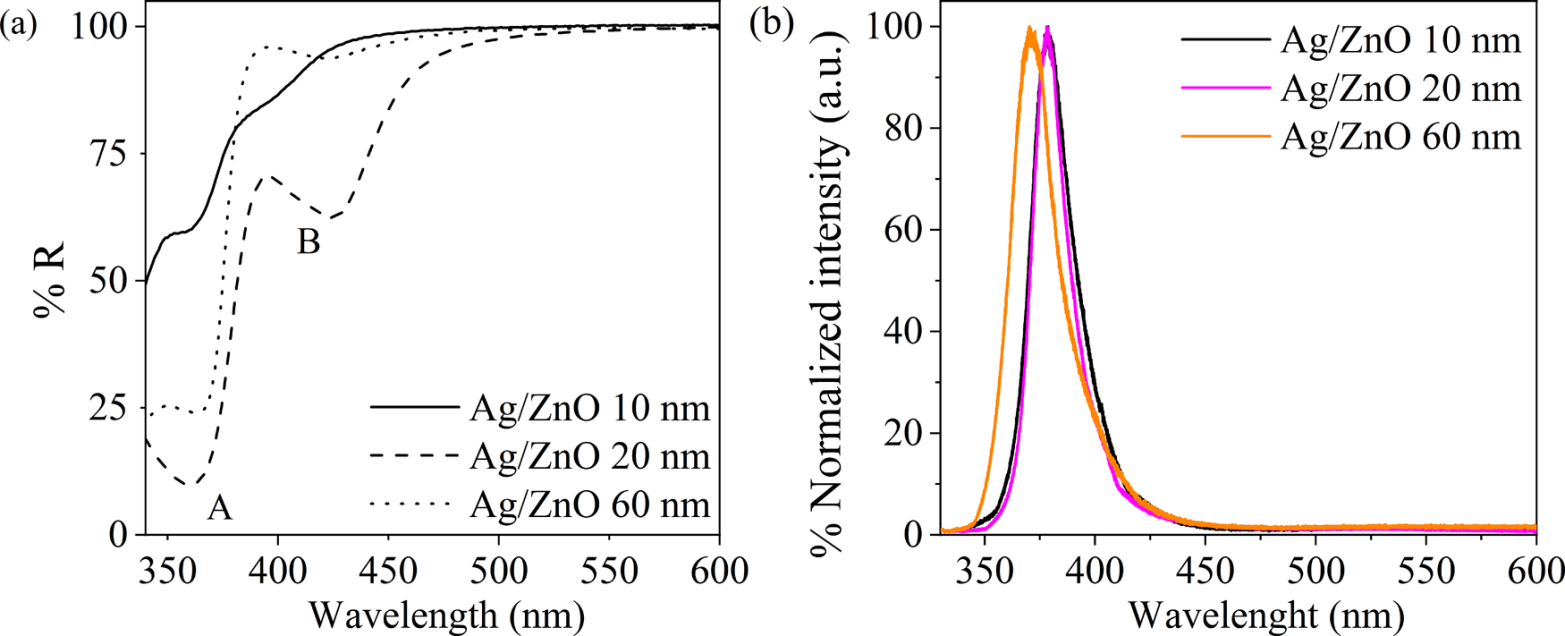
(a) Diffused reflectance spectra of Ag/ZnO with various thicknesses. The two reflection bands at ∼362 nm (A) and ∼390–414 nm (B) are assigned to absorption. (b) PL spectra of Ag/ZnO with various thicknesses with an excitation wavelength of 325 nm. The near-band-edge emission present between 378 and 370 nm is associated with band-to-band radiative recombination. No emission band was detected above 500 nm.

We deposited ultrathin ZnO layers on Ag by ALD. Two absorption bands are evident in the UV–vis reflection spectra of Ag/ZnO (Figure 1a). Band “A” at ∼362 nm stems from the valence to conduction band electronic absorption in ZnO, and band “B” at ∼390–414 nm stems from the interaction of Ag and ZnO and is not apparent in the individual (separated) materials (Figure S1).

While band A has an onset and maximum absorption wavelengths (minimum reflection) that only slightly change with the ZnO thickness, the onset and position of band B vary significantly with ZnO thickness. Also, the absorption maximum of band B red-shifts when the thickness of ZnO is increased from 10 to 20 nm, while its intensity diminishes when the ZnO thickness is increased from 20 to 60 nm. Band B in the 20 nm thick ZnO layer has the most extended absorption tail, the widest absorption peak, and the largest absorption intensity among all of the three thicknesses we tested.

The common defect states in ZnO, such as oxygen vacancies or sodium/lithium substitutional impurities (that could diffuse from the glass substrate), are known to result in a green or yellow emission above 500 nm.^18,28,60,61^ However, the PL spectra of Ag/ZnO in the 330–600 nm region (Figure 1b) show only a near-band-edge emission and complement the band-to-band absorption band A. This can imply that the ZnO films lack common defects, such as oxygen vacancies or sodium/lithium substitutional impurities, or, more likely, that an additional mechanism quenches the emission. Additionally, the maximum PL position is slightly blue-shifted from 378 to 370 nm when the ZnO thickness is increased from 20 to 60 nm. The band gap of bulk ZnO at room temperature was previously reported to be at 378–381 nm (based on the maximum PL peak energy),^18^ which implies that the Ag/ZnO 60 nm layer has a slightly larger effective band gap compared to the 10 and 20 nm Ag/ZnO layers or to bulk ZnO. We discuss these effects further below (*vide infra*).

**Figure 2:**
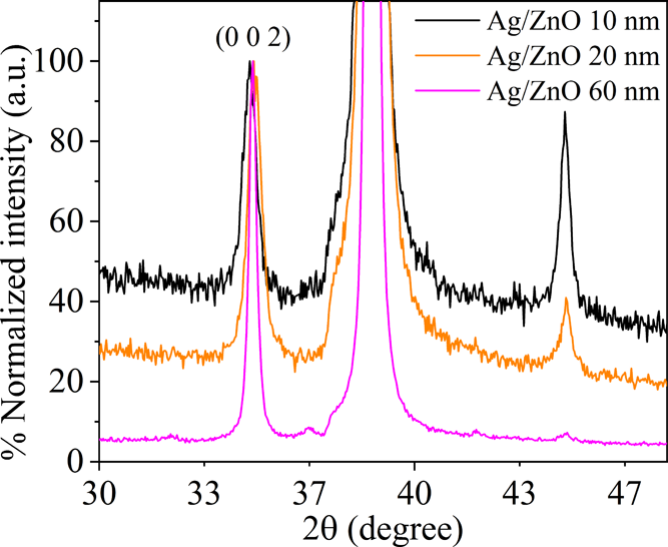
XRD patterns of Ag/ZnO with various ZnO thicknesses. The diffraction pattern matches a wurtzite ZnO and fcc Ag. The diffraction peak at ∼34.8° stems from the (002) plane of ZnO. This peak becomes narrower and shifts to higher 2θ values in thicker films, indicating larger grains and a higher residual stress in thicker ZnO films. The diffraction peaks at 38.6 and 44.7° stem from the Ag.

The variation of XRD patterns of the Ag/ZnO films with different ZnO thicknesses is shown in Figure 2. The peaks at ∼34.8, 32.3, and 36.6° stem from the (002), (100), and (101) planes of wurtzite ZnO (ICSD file no. 26170; the latter two are evident only in thicker ZnO films), while the peaks at 38.6 and 44.7° belong to Ag face-centered cubic (fcc) (ICSD file no. 44387).

In our films, the diffraction peak at 34.8° has the highest intensity, while in powder diffraction, the peak at 36.6° has the highest intensity. This difference indicates a preferred orientation of the (002) plane with respect to the substrate. Also, the (002) peak shifts to higher 2θ values in thicker ZnO films, while the peaks associated with Ag are constant. The width of the peak at 34.8° becomes narrower with the 60 nm film, which indicates smaller crystallites in the thinner films than in the thicker ones.^20^ Lastly, the shift in the 34.8° peak position towards higher 2θ values indicates the presence of a compressive strain in the films, which is more prominent in the thicker films. For the 10 and 20 nm films, the small grains can reduce the lattice strain compared to the 60 nm film. Effective expansion of the ZnO band gap can be caused by lattice strain and could explain the observed blue shift in the PL peak of the 60 nm thick films.^62^

**Figure 3:**
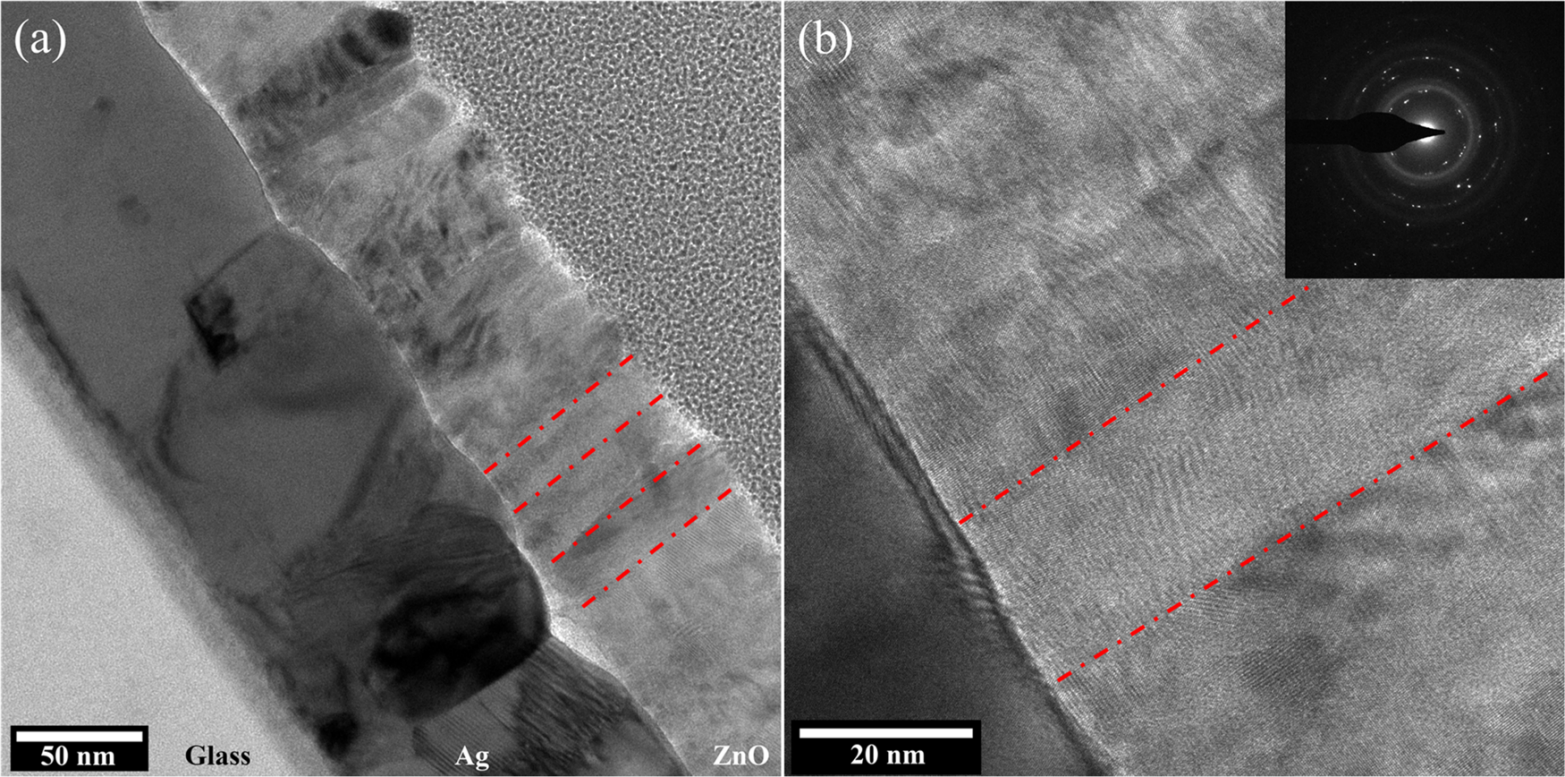
TEM images of the Ag/ZnO 60 nm (a) 120k× magnification. The ZnO layer exhibits columnar grains oriented perpendicular to the surface. (b) 650k× magnification. The columns start to grow from the Ag/ZnO interface. Inset: selected area electron diffraction (SAED) pattern for the hexagonal wurtzite ZnO film.

The preferred orientation of the crystallites is also discernible in electron microscopy images of the cross sections of the Ag/ZnO layers. Figure 3a shows that the ZnO film has columnar grains with an average width of ∼15 nm, oriented perpendicular to the substrate. A close look at the Ag/ZnO interface (Figure 3b) manifests that the columns tend to grow from the Ag/ZnO interface upward, with no discernable amorphous or granular layer near the interface. The selected area electron diffraction (SAED) pattern in the inset of Figure 3b matches the diffraction pattern of wurtzite ZnO, and the signal associated with the (100) plane has the highest intensity, which also indicates a preferred crystalline orientation (see also the data in Figure S3 and Table S1 in the Supporting Information, SI).

**Figure 4:**
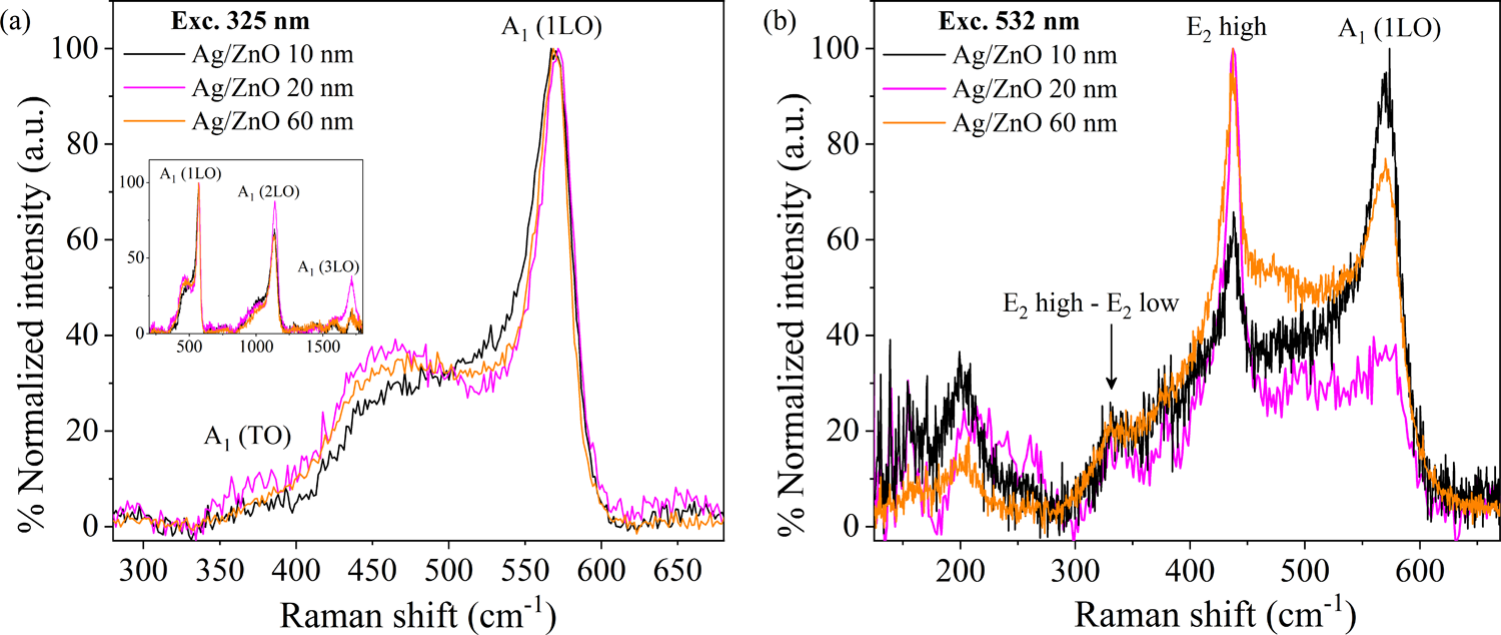
Raman spectra of Ag/ZnO with various ZnO thicknesses. (a) Resonant Raman spectra with an excitation wavelength of 325 nm. Inset: broader spectrum range, dominated by multiple A_1_ (LO) peaks at 571, 1140, and 1720 cm^–1^. (b) Nonresonant Raman scattering spectrum with an excitation wavelength of 532 nm, dominated by the E_2_ high mode at 437 cm^–1^ and A_1_ 1LO at 570 cm^–1^. (Non-normalized Raman spectra are presented in Figure S2).

We used Raman spectroscopy to examine the ZnO defects and chemical bonding at the Ag/ZnO interface. The resonant Raman scattering spectra of Ag/ZnO in the 200–1800 cm^–1^ region are dominated by multiple peaks at 571, 1140, and 1720 cm^–1^ (Figure 4a) that mainly stem from A_1_ (LO) vibrations along the *c*-axis of the hexagonal ZnO crystal structure—specifically, from vibrations along the Zn–O polar bonds that are oxygen vibrations centered.^18,63,64^ Some mixing of E_1_ (LO) in these bands is possible,^64,65^ but the minor shift (<3 cm^–1^) of the A_1_ (LO) band relative to the reported A_1_ (LO) band of single crystals of ZnO indicates that the bands are mostly the A_1_ (LO).^65^ The 1140 and 1720 cm^–1^ bands are higher-order scatterings of A_1_ (LO).

Unlike the Raman spectrum of ZnO wurtzite single crystals,^65^ the A_1_ (LO) peaks in our thin films are accompanied by a shoulder. For example, the A_1_ (LO) at 571 cm^–1^ is accompanied by a shoulder at ∼470 cm^–1^. Also, a small peak centered near ∼380 cm^–1^ is discernable. The shoulder at ∼470 cm^–1^ was previously assigned to surface optical phonon modes with A_1_ symmetry (SO),^63^ while the shoulder at ∼380 cm^–1^ was assigned to a confined A_1_ (TO) mode.^66^ The A_1_ (LO) band (571 cm^–1^) in the 10 nm ZnO film is broader than in thicker films, and the intensity of the SO band (470 cm^–1^) is higher in the 20 and 60 nm thick films than in the 10 nm thick film. Lastly, the A_1_ (TO) band (∼380 cm^–1^) has a slightly higher intensity in thicker films.

The A_1_ (LO) band (571 cm^–1^) in the 10 nm thick sample is broader than in the thicker films, indicating a broader distribution of bond lengths and smaller crystallites in the 10 nm thick sample, in agreement with our XRD results. The ratio of SO to LO bands’ intensity correlates with the surface-to-volume ratio of ZnO, i.e., with the density of surface defects.^63^ The SO/LO ratio is highest for the 20 nm thick film, which indicates that it has the highest density of surface defects, which correlates with the absorbance in the UV–vis band B being the highest at 20 nm. Finally, the A_1_ (TO) band intensity is sensitive to the wurtzite crystal orientation with respect to the surface,^18^ and the slightly higher intensity in the thicker films indicates that the ZnO grains are more (preferentially) oriented in the thicker films.

The nonresonant Raman scattering spectrum is dominated by the E_2_ high mode at 437 cm^–1^ and A_1_ (1LO) at 570 cm^–1^ (Figure 2b). A small band at ∼340 cm^–1^ is assigned to the second-order Raman band arising from differences between E_2_ high and E_2_ low vibrations.^18^ This vibration band has a similar intensity in all films. Another small band at ∼200 cm^–1^ with a higher intensity for the thinner ZnO films is apparent. We note that for Ag/ZnO 10 and 20 nm, the A_1_ (LO) band intensity follows an inverse trend compared to the E_2_ high band intensity. We detected no Raman band in this region for Ag (Figure S2).

The E_2_ high band was previously associated with the crystallinity of ZnO.^28,61,67^ The increased E_2_ peak intensity in the thicker ZnO films suggests that thicker films are more crystalline than thinner ones, which is in agreement with previous studies of crystallinity of ALD-deposited films^68^ and agrees with our interpretation of the broader A_1_ (LO) band in thinner ZnO films and with our XRD results.

As reflected in the XRD and TEM results, all of the ZnO films have a preferred orientation in the *c*-axis direction with respect to the surface that affects the amplitude of polar vibrations near the interface. E_2_ high and A_1_ (LO) modes are both oxygen-dominated, but the E_2_ high mode shows a nonpolarized oscillation perpendicular to the *c*-axis, while the A_1_ (LO) mode has a polarized oscillation along the *c*-axis.^18^ The higher intensity of the A_1_ (LO) mode and the lower intensity of the E_2_ high mode in the 10 nm thick films are attributed to a dipole (supported by the XPS analysis below) at the Ag/ZnO interface that enhances the amplitude of polarized vibrations perpendicular to the surface.

Lastly, local vibration modes (LVM) were previously ascribed to Ag substitutional defects in ZnO at 418 and 244 cm^–1^;^69^ based on this, we tentatively ascribe the band at ∼200 cm^–1^ to interlayer bonds, Ag–O–Zn, that form at/near the interface of the heterostructure and the wavenumber shift to the different chemical environment between Ag substitutional defects in ZnO and Ag–O–Zn at the Ag/ZnO interface.

**Figure 5:**
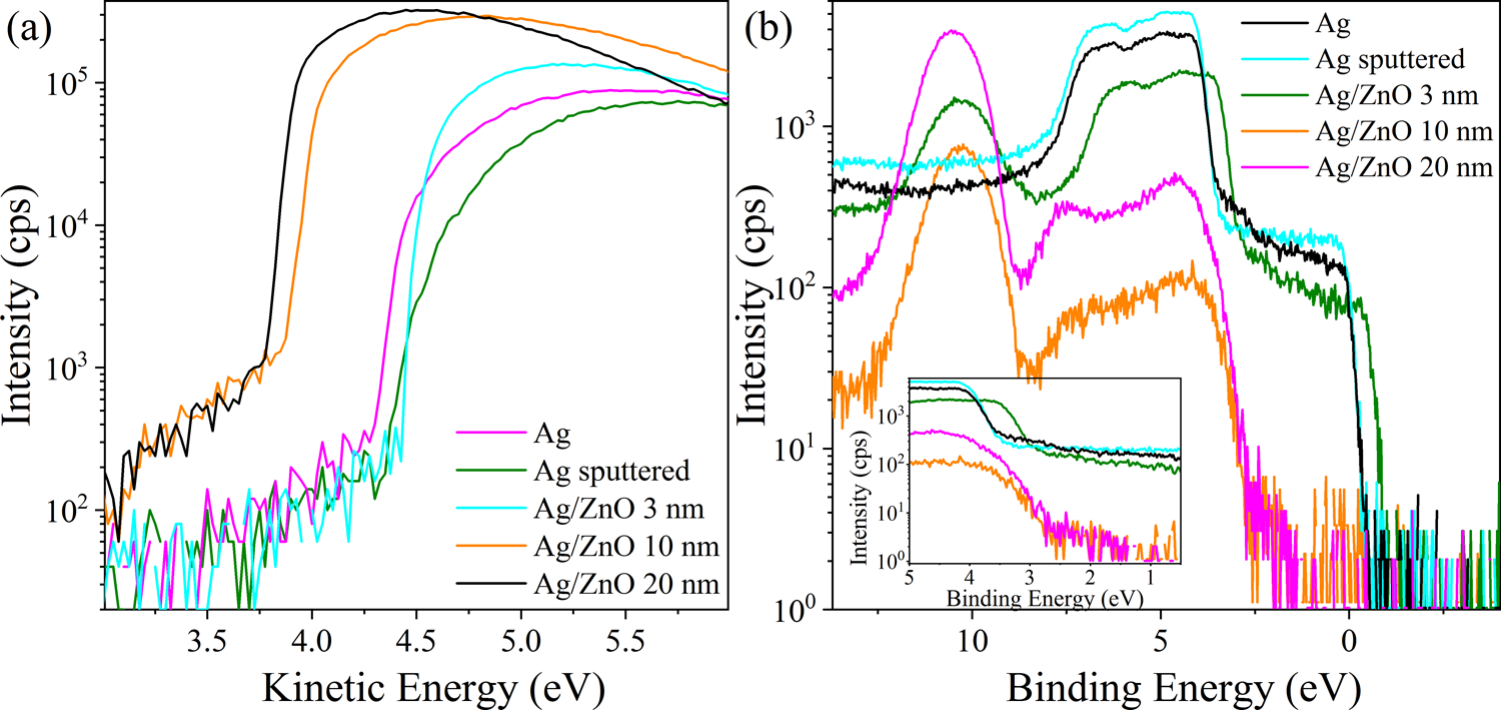
(a) Secondary electron emission (SEE) spectra of Ag, Ag/ZnO 3, 10, and 20 nm. The spectroscopic onsets (lowest kinetic energy) designate the samples’ work function. (b) Valence band photoelectron spectra of Ag, Ag/ZnO 3, 10, and 20 nm, already corrected for charging effects; see the Experimental Section for details. Inset: Top valence band region.

To portray the energy-band diagram and analyze the Ag/ZnO interface chemistry, we carried out XPS measurements, from which the top of the valence band and the work function of the samples could be extracted, in addition to the standard XPS compositional and oxidation states analysis. Figure 5b presents the valence band spectra of the corresponding samples. Reliable values for the valence band maximum (VBM) energy for the 10 and 20 nm layers can thus be extracted. However, overlap with the silver bands introduces practical difficulties with the 3 nm specimen. First, the charging correction applied here was evaluated for ZnO, and since the silver was practically well grounded, an off-E_F_ edge of its conduction band was artificially introduced. Second, we suspect that the interface field in thinner films degraded faster under the X-ray irradiation than in thicker films. Altogether, VBM values in the range of 2.70–2.73 eV were derived from this analysis (corrected for the charging artifacts). Note that the top valence band edge of Ag/ZnO 20 nm shows a “long” tail of states up to 1.73 eV above the valence band maximum, indicating the presence of band-gap defect states, which we associate with zinc hydroxide.^70^

Work-function (WF) measurements corrected for charging artifacts are presented in Figure 5a.^59^ Apparently, the ZnO layer tends to reduce the WF, leading to WF values about 0.5 eV smaller than that of the silver, as seen in Figure 5a for the 10 and 20 nm thicknesses. Importantly, for 3 nm ZnO, we found an increase in WF compared to both the substrate itself (more than 0.1 eV; before or after sputtering) and thicker ZnO layers. This result is supported by the measured Zn 2p_3/2_ peak positions: 1021.67, 1021.99, and 1022.02 eV, for 3, 10, and 20 nm of ZnO,^71^ respectively; the values were already corrected for charging artifacts. In general, the latter differences may reflect changes in the Zn oxidation state, but can also reflect differences in the mean electrostatic potential (e.g., due to distant charges). Combined with the measured WF variations, we conclude that the latter cannot be excluded, and an interface dipole does exist in this system.

Our compositional analysis suggests thickness-dependent stoichiometry variations in the ZnO layer. To start with, we find a considerable amount of zinc hydroxide in these samples. On top of that, the 3 nm layer is slightly oxygen-deficient (based on values close to the level of experimental error), O_L_/Zn = 0.96, while the 10 and 20 nm layers are slightly oxygen-rich, with O_L_/Zn = 1.02, where O_L_ stands for the mixed stoichiometry associated with the presence of hydroxide within the ZnO matrix (see Table S2 in the SI). The hydroxide content (a shoulder in the O 1s line) is minimal in the 10 nm sample, which suggests that zinc hydroxide is distributed nonuniformly, realizing higher concentration levels both near the Ag/ZnO interface and near the top ZnO surface.

**Figure 6:**
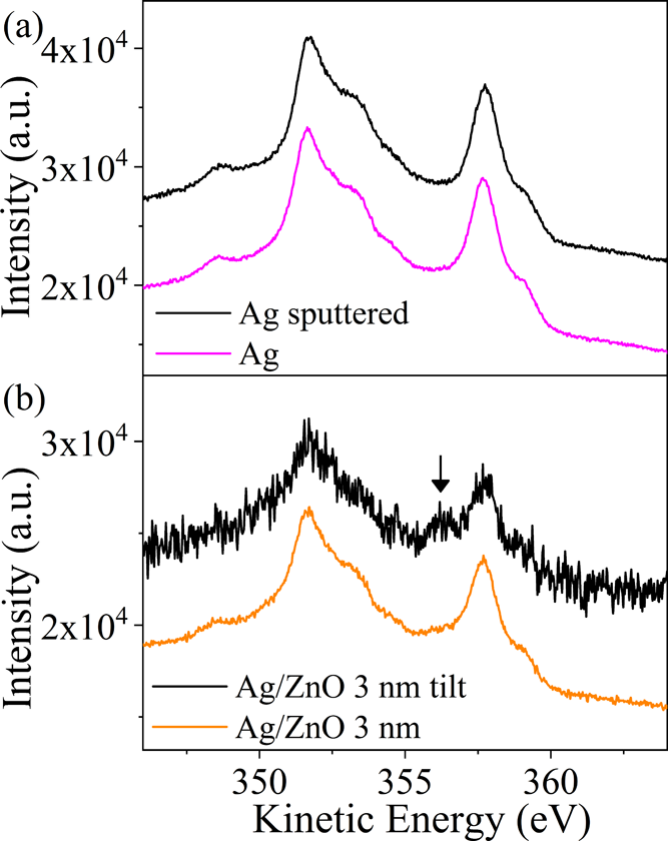
Ag M_4_N_4,5_N_4,5_ spectrum as measured from (a) bare Ag, before and after Ar sputtering, and (b) Ag/ZnO 3 nm at emission angles of 0 and 60°. Nearly no changes are seen in the M_4_N_4,5_N_4,5_ spectrum, except for the small peak at 356.3 eV in the Ag/ZnO 3 nm sample at the grazing angle configuration, corresponding to Ag*_x_*O.

Finally, we would like to inspect the interface chemical bonds. The (X-excited Auger) Ag M_4_N_4,5_N_4,5_ spectrum is shown in Figure 6, for bare Ag, Ar-sputtered Ag, and the Ag/ZnO 3 nm sample at normal (0°) and grazing (60°) emission angles. For Ag (bare or Ar-sputtered), no peaks corresponding to silver oxide were found, while after deposition of 3 nm of ZnO, a small peak at 356.3 eV appeared (better seen in the grazing angle configuration at 60°, namely under enhanced surface sensitivity). No indication for this peak was found in the thicker films, a fact that might reflect the limited depth sensitivity at this energy range. We attribute this peak to Ag_2_O,^72,73^ also supported by a small tail at 367.5 eV in the Ag 3d line and an ∼30 meV shift of its peak position, from the binding energy (BE) of 368.28 to 368.25 eV (not shown).^74^ These latter differences suggest that Ag*_x_*O is formed at the interface when ZnO is deposited.

**Figure 7:**
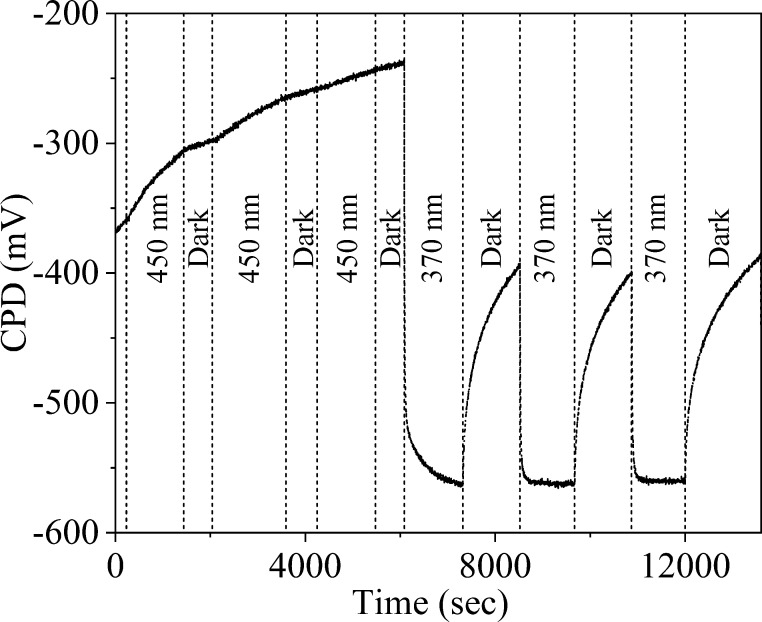
Photo-CPD response of Ag coated with 60 nm of ZnO. Each vertical dotted line designates a transition of the lighting conditions (dark, 450, or 370 nm)—the contact potential difference changes in opposite directions with the response to 450 or 370 nm light. The transients to stabilization also have different time scales. Measurements were done in a faraday cage with LED lighting in ambient air.

To investigate the photovoltaic response of the metal–metal oxide junction, we measured the light-induced changes in contact potential difference (CPD) for the various thicknesses. The CPD changed from ∼300 to ∼−120 and from ∼−230 to ∼−370 mV by depositing 10, 20, or 60 nm thick ZnO on Ag (not shown), which indicates that the work function of ZnO is smaller than the work function of Ag and that electrons are transferred from ZnO to Ag when the contact is formed (in agreement with the XPS-based SEE analysis). Interestingly, however, the surface photovoltage (Figure 7) showed unusual characteristics under illumination with sub- and supra-band-gap photons.

When illuminated with a 370 nm light (supra-band gap), the CPD changed to more negative values (ΔCPD < 0) and reached a constant value after approximately 2 min. Nevertheless, when the 370 nm light was switched off, the CPD values did not reach a fixed value even after 20 min. When the samples were illuminated with a wavelength of 450 nm (sub-band-gap), the CPD slowly changed to less negative values (ΔCPD > 0), but did not stabilize at a constant value even after 20 min. When the 450 nm light was switched off, no prompt response was found at all, and the CPD remained constant for tens of minutes. Excitation at even longer wavelengths (700 nm) did not yield any observable surface photovoltage, whereas excitation with white light, which covered both the 370 and 450 nm regimes, resulted in ΔCPD < 0. Lastly, shining light on Ag without ZnO yielded no surface photovoltage, and Ag/ZnO samples with a thinner ZnO layer (10 and 20 nm) showed a qualitatively similar photoresponse to 370 and 450 nm wavelengths, albeit with a smaller amplitude (Figure S5a,b, respectively). Additional experiments within the XPS chamber gave consistent results for the response to light, as examined by the XPS-based WF measurements.

## Discussion

Thin layers of ZnO with preferred crystal orientation were deposited on Ag by ALD and resulted in the formation of an optical absorption band that stems from the interface band B in the UV–vis spectrum. This transition is absent from the separated materials, and its energy rules out known defect states in ZnO. Furthermore, plasmonic excitations are also unlikely to be the cause for this optical band since opposite directions in the CPD response are observed for all different ZnO thicknesses under illumination with sub-band-gap (exciting band B) and supra-band-gap (band-to-band excitation) photon energies. Therefore, we ascribe this band to an electronic transition and discuss its physical origin.^75^

**Figure 8:**
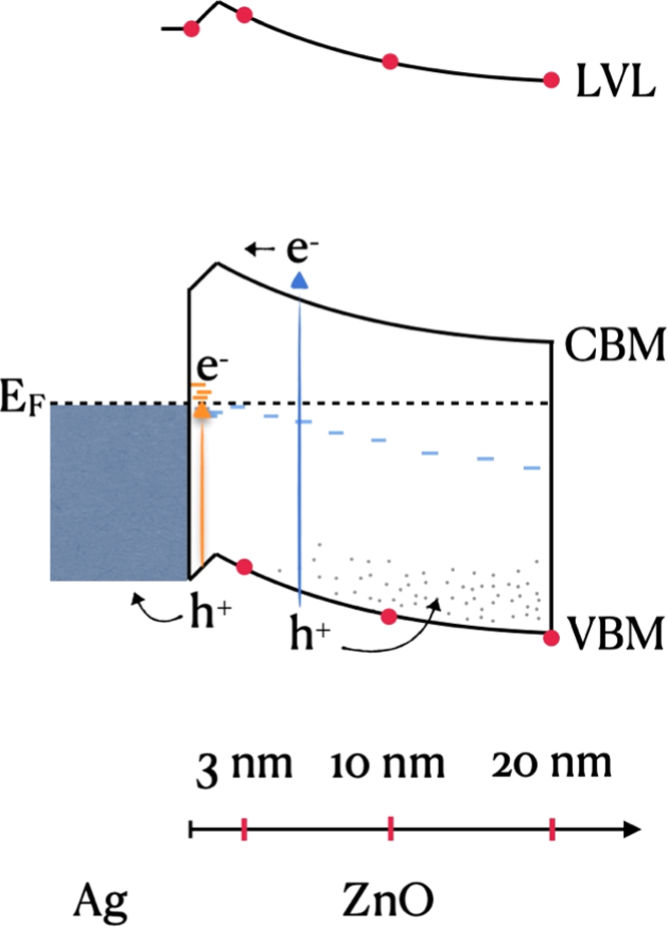
Energy-level diagram of Ag/ZnO, as extracted in reference to the Fermi level (black dashed line). VBM, CBM, and LVL stand for valence band maximum, conduction band minimum, and local vacuum level, respectively. Red dots designate values directly extracted from measurements, whereas the band profiles (black lines) indicate interpolation between the measured data points. The VBM to CBM energy difference is set equal to the optical band gap of ZnO. Defect states associated with oxygen vacancies are illustrated as light-blue lines ∼0.7 eV below the CBM (based on previous reports). Hole traps associated with zinc hydroxide near the surface extend from the VBM into the band gap; the illustration does not represent a specific structure. An electrostatic potential upshot is apparent near the interface, involving oxygen vacancies; hence, deep states are available for electron trapping. Sub-band-gap excitation (orange lines and arrow at the Ag/ZnO interface) causes electron trapping at the interface, while holes are transported to the ground through the Ag contact. Supra-band-gap excitation (blue arrow) does not flatten the bands because the holes are captured by trap states associated with zinc hydroxide mainly enriched at the top surface (illustrated as gray dots; electrons are extracted to the ground through the Ag contact).

Based on photoelectron spectroscopy, an energy-band diagram was constructed, as illustrated in Figure 8. The diagram resembles the Schottky junction of an n-type semiconductor in contact with a metal of a larger work function, as compared to that of the semiconductor.^27^ However, the decrease in CPD during supra-band-gap illumination is contrary to the expectation from the built-in electric field (Figure 8). The latter would suggest photo-induced electron accumulation at the ZnO surface, whereas the actual result corresponds to hole accumulation. In addition, the increase in CPD under sub-band-gap illumination implies that electrons accumulate (and/or holes are annihilated) within the ZnO layer and/or its inner interface. We attribute these nontrivial observations to the presence of two dominant types of defect states, one for electrons and the other for holes. Each type is dominant at different spatial locations within the heterostructure. The dominance of one trapping mechanism over the other is also affected by the illuminating wavelength. As a result, sub- vs supra-band-gap SPV response is realized, as explained hereafter.

Previously, oxygen vacancies near the metal/ZnO interface were proposed to introduce interface defects ∼0.7 eV below the conduction band minimum due to chemical bonds formed between the metal and molecular oxygen near the interface, thus resulting in electron transfer to the metal, ionization of the vacancies, and Fermi-level pinning. Our XPS and Raman results confirm independently that the ZnO is oxygen-deficient near the Ag/ZnO interface, in agreement with previous reports. This feature is further supported by the interface Auger peak assigned to Ag*_x_*O. We also suggest that the Ag*_x_*O–Zn bonds at the interface provoke a local vibration mode, similar to previous reports on a “local vibration mode” that was provoked when Ag was incorporated in the ZnO wurtzite crystal structure.^69^ Here, the Ag ions’ symmetry and the chemical environment of the interface Ag*_x_*O–Zn bonds are different, which leads to a shift from ∼244 to ∼200 cm^–1^ in the vibration band position. Effectively, the formation of Ag*_x_*O–Zn implies an excess of oxygen vacancies near the interface. Yet, we are not aware of any literature report on optical transition associated with these interface states, nor with oxygen vacancies in bulk ZnO. Furthermore, the presence of oxygen vacancies by itself is insufficient to explain other experimental observations, such as the ‘thickness-dependent’ absorbance maxima.

Analysis of the interface energy levels and the electrostatic potential variations reveals an explanation consistent with the optical data and its related thickness dependence. We first note that the E_F_ level is located 2.75 eV above the VBM, which meets the photon energy used for sub-band-gap illumination and coincides with the energy-level position of the oxygen vacancies. Next, we suggest that the local increase in WF, evident in the 3 nm thick ZnO, reflects a significant interface dipole, probably restricted to the <3 nm region, which is effectively rich in oxygen vacancy states (∼0.7 eV from the CBM). The E_F_ level and the local increase in WF, taken together, imply that the near-interface oxygen vacancies (at least a portion of those) lay above the Fermi level and, therefore, are not populated at equilibrium by electrons. This attribute makes these defect states available for accepting and trapping photoexcited electrons from the VBM, i.e., optically active.

Finally, the local potential varies with the thickness of the ZnO layer as the electronic junction is formed. This is evident in the WF variation with ZnO thickness and is possibly explained by the small electrostatic screening ability of ultrathin layers of a semiconductor due to the lower availability of mobile charges. Since we did not find any indication for Ag ions that diffused into the ZnO layer and introduced oxygen vacancies at “deeper” locations in the native ZnO, we conclude that farther from the interface, the concentration of oxygen vacancies is lower than near the interface (a result also supported by the XPS analysis). Yet, due to the variation in local potential upon junction construction, the occupancy of the interface defect states varies with the thickness of the ZnO layer. This explains the ‘thickness dependence’ of band B (intensity and position). To complete the picture, we ascertain that holes generated by sub-band-gap illumination can accumulate near the interface. Their proximity to the metal necessarily dictates rapid transport to the Ag substrate, thus leaving a net negative charge trapped near the interface. This assertion of charge separation is supported by the lack of photoluminescence emission in the visible range and observed SPV data.

Alternatively, photogenerated holes can be trapped in hole traps associated with Zn-hydroxide. These hole-trap states reside throughout the entire ZnO, but are found to be enriched near the top surface. Under supra-band-gap illumination, hole trapping becomes a dominant process as it occurs throughout the entire ZnO layer (and more so near the top surface). Therefore, despite the internal (dark) electrostatic field that tends to dictate electron accumulation at the surface, the response to supra-band-gap light is dominated by the available hole traps, and thus, nontrivial SPV response is realized.

Two effects are suggested to contribute to the formation of the interface dipole: (1) a molecular-like dipole across the Ag–O–Zn bonds, and (2) the native ZnO lattice polarity, for which the preferred orientation (evident from the XRD and TEM results) implies long-range contributions. In any case, the Ag–O–Zn bonds formed at the interface experience a large local electric field. A similar interface dipole was found at the TiO_2_/Au interface, introducing intriguing implications on the catalytic properties of this construct. The Ag*_x_*O–Zn interface states likely have a long lifetime, as indicated by the long stabilization times of the SPV signal, which can be useful for driving photoelectrochemical reactions. Their temporal characteristics may be studied in more detail by, e.g., transient absorption spectroscopy, and their structure can be further investigated by synchrotron methods, such as EXAFS and NEXAFS, or solid-state NMR. Such studies can potentially promote the utilization of the bifunctional photoresponse observed here for photocatalysis, or the development of passivation strategies that facilitate metal/metal oxide electronics.

## Conclusions

We investigated the optoelectronic properties and chemical structure of the Ag/ZnO heterostructure and its interface in particular. An optical transition, not reported previously, was revealed, assigned to deep energy states (near E_F_) at the interface. We conclude that this transition evolves from the Ag–O–Zn interface chemical bonds, involving a substrate-overlayer charge transfer that effectively introduces (1) oxygen deficiency mainly near the interface and (2) a nonmonotonic variation in the energy bands. These two effects partly empty the interface states and make them optically active to sub-band-gap illumination. A second type of “defects” was found as well, associated with the presence of zinc hydroxide that tends to be enriched at the top surface of the film. These defects act as hole traps and dominate the surface photovoltage response under supra-band-gap illumination. Overall, the presence of two types of electronically active traps, combined with an interface dipole, results in opposing photovoltaic responses, depending on the excitation wavelength. The proposed new insight into the Ag/ZnO interface structure and its consequent optoelectronic properties can be used in designing photocatalysts or metal/metal-oxide electronics.

## Supporting Information Available

UV–vis transmission measurement of ZnO, non-normalized Raman spectra, SAED pattern, and data for the hexagonal wurtzite ZnO film, XPS data, SPV response of Ag/ZnO 10 and 20 nm (PDF)
