# Supplementary material for: Chemistry and Charge Trapping at the Interface of Silver and Ultrathin Layers of Zinc Oxide
Source: ACS Appl Mater Interfaces. 2021 Oct 9;13(41):49423–32. doi: 10.1021/acsami.1c11566 (PMC8592502; doi:10.1021/acsami.1c11566)
Supplement: Supplementary file 1 — am1c11566_si_001.pdf [file am1c11566_si_001.pdf]

Supporting Information

Chemistry and Charge Trapping at the Interface of Silver and Ultra-Thin Layers of Zinc Oxide

M. Rahamim,[1] H. Cohen,[2] E. Edri[1,3,4*]

[1]Department of Chemical Engineering, Ben-Gurion University of the Negev, Be'er-Sheva 8410501, Israel, [2]Department of Chemical Research Support, Weizmann Institute of Science, Rehovot 7610000, Israel, [3]Ilse Katz Institute for Nanoscale Science and Technology, Be'er-Sheva 8410501, Israel, [4]Blechner Center for Industrial Catalysis and Process Development, Beer-Sheva 8410501, Israel

*Corresponding author email: edrier@bgu.ac.il

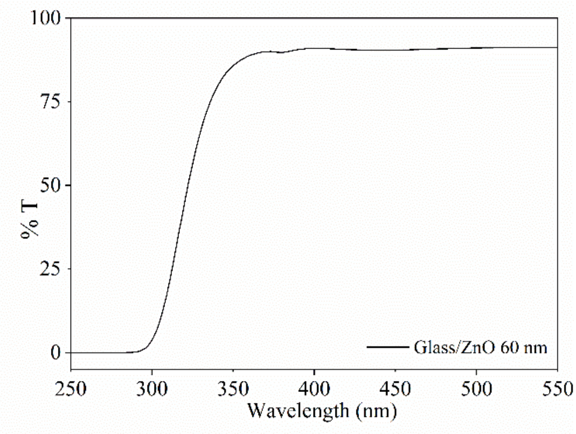

Figure S1. UV-vis transmission measurement of 60 nm of ZnO deposited on top of a clean glass slide. UV-vis transmission measurement was carried out instead of reflectance due to the transparent ZnO film.

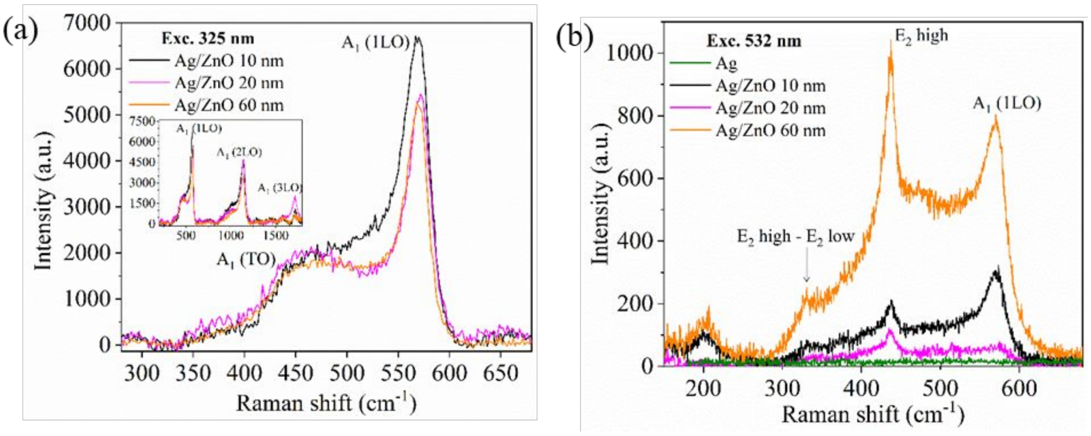

Figure S2. Non-normalized Raman spectra of Ag/ZnO with various ZnO thicknesses. (a) Resonant Raman spectra with excitation wavelength of 325 nm. Inset: broader spectrum range, dominated by multiple A_1 (LO) peaks at 571, 1140, 1720 cm^{-1}. (b) Non-resonant Raman scattering spectrum with excitation wavelength of 532 nm, dominated by E_2 high mode at 437 cm^{-1} and A_1 1LO at 570 cm^{-1}.

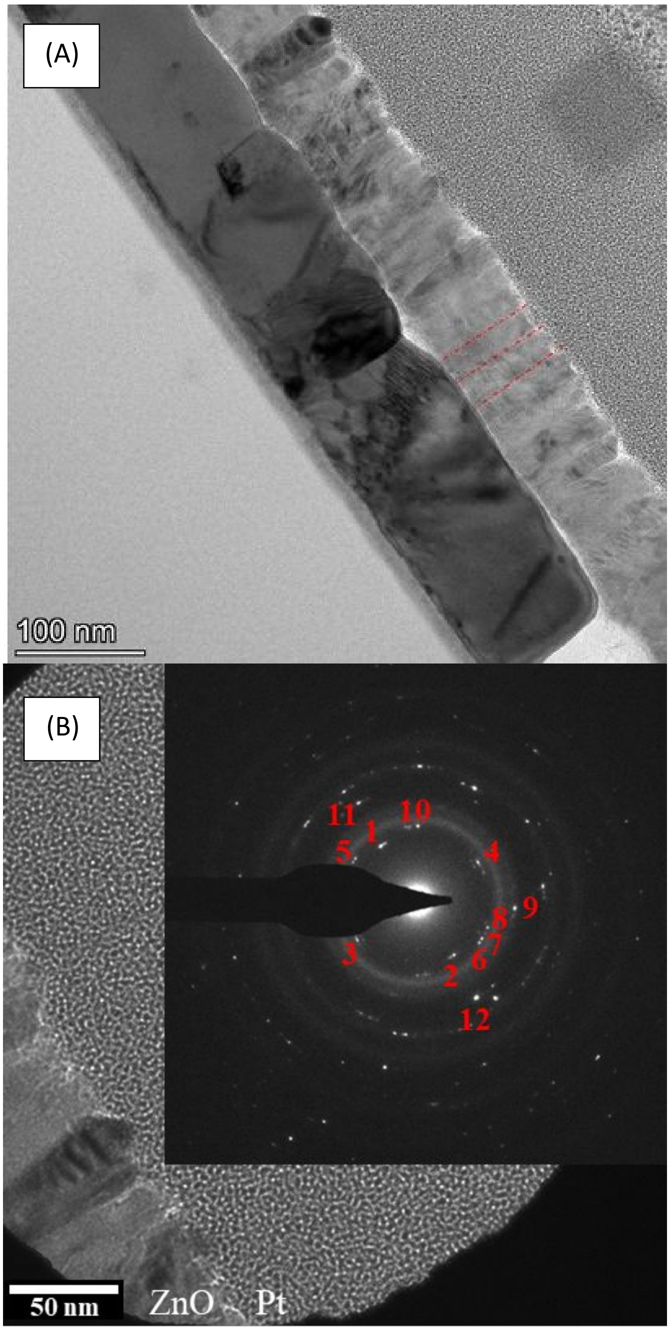
(A)
100 nm
(B)
11
1
10
5
4
9
8
7
6
3
2
12
50 nm
ZnO
Pt

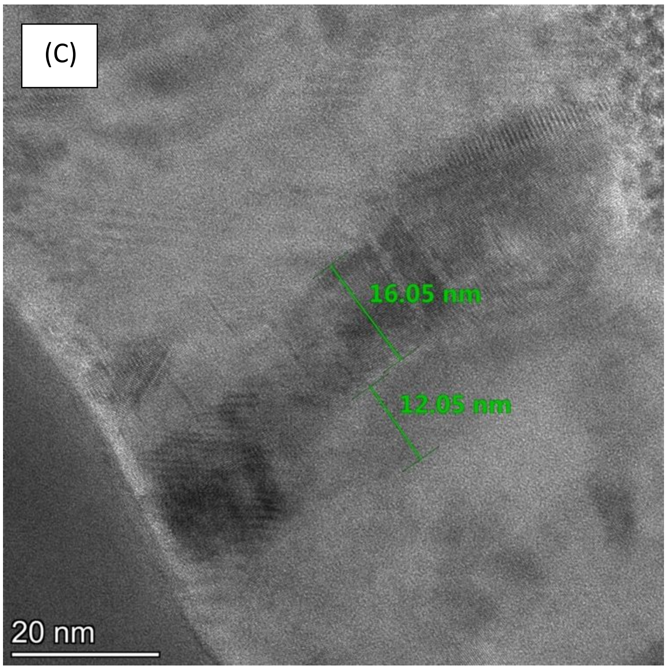

Figure S3. (A) TEM images of the Ag/ZnO 60 nm X120k. The columnar growth of the ZnO crystallites is discernable. The red dash-dot lines designate two columns. (B) selected area electron diffraction (SAED) of the Ag/ZnO 60 nm focused only on the ZnO film. Insert: The SAED pattern match the hexagonal wurtzite ZnO film. (C) TEM images of the Ag/ZnO 60 nm X650k with the width of two ZnO columnar grains marked.

Table S1. SAED pattern analysis of the ZnO film as shown in the inset of Figure S3.

SPOT NO.	D-SPACING (NM)	REC. POS.(1/NM)	DEGREES TO SPOT 1	DEGREES TO X-AXIS	AMPLITUDE	PLANE
1	0.2869	3.485	0	123.92	2835	1.0.0
2	0.2869	3.485	180	-56.08	1399	1.0.0
3	0.2616	3.822	90.08	-146.01	470	0.0.2
4	0.2625	3.81	91.65	32.27	904	0.0.2
5	0.2516	3.974	21.79	145.71	469	1.0.1
6	0.2509	3.985	158.34	-34.43	703	1.0.1
7	0.2509	3.985	154.88	-30.97	669	1.0.1
8	0.2464	4.058	147.02	-23.1	110	1.0.1
9	0.1921	5.206	128.86	-4.94	493	1.0.2
10	0.2523	3.964	34.9	89.02	443	1.0.1
11	0.1652	6.054	2.95	120.97	637	1.1.0
12	0.1653	6.049	176.82	-59.26	2411	1.1.0

Table S2. Raw atomic concentrations (%) for bare Ag, before and after sputtering, and Ag/ZnO 3, 10, and 20 nm.

Sample	***Ag after sputtering***	***Ag before sputtering***	***Ag/ZnO 3 nm***	***Ag/ZnO 10 nm***	***Ag/ZnO 20 nm***
Ag 3d (368.2)	100	58.67	18.14	0.74	0.02
Zn 2p (1021.9)	-	-	18.10	25.45	22.98
O 1s $_{(M)}$ ***(531)***	0	5.29	13.62	20.15	18.36
O 1s $_{(OH)}$ ***(532.5)***	0	0.48	7.80	8.66	10.36
C 1s $_{(C)}$ ***(~285)***	0	32.32	25.27	17.80	22.23
C 1s $_{(ox)}$ ***(~288)***	0	3.24	2.84	1.41	1.99
Ratio $(O_L)/Zn2p$	-	-	0.96	0.96	1.02
Ratio Zn3p/Zn2p	-	-	0.78*	1.01	1.04

The $_{(M)}$ and $_{(OH)}$ indexes of O 1s correspond to oxygen in metal oxide and hydroxide bonds, respectively. The $_{(C)}$ and $_{(ox)}$ indexes of C 1s correspond to carbon in the C-C/C=C bonds and C=O/C-O bonds, respectively. The O_L:Zn ratio was calculated according to $O_L = O1s_{(M)} + (0.5 * O1s_{(OH)})$.

*As a cross-check, the Zn3p/Zn2p was evaluated, yielding =1 at high accuracy (within the experimental error) for both the 10 and 20 nm samples. For the 3 nm sample, a technical difficulty was encountered, due to overlap of signals that introduced a significant error in background subtraction, thus raising the relative experimental error to about 40%.

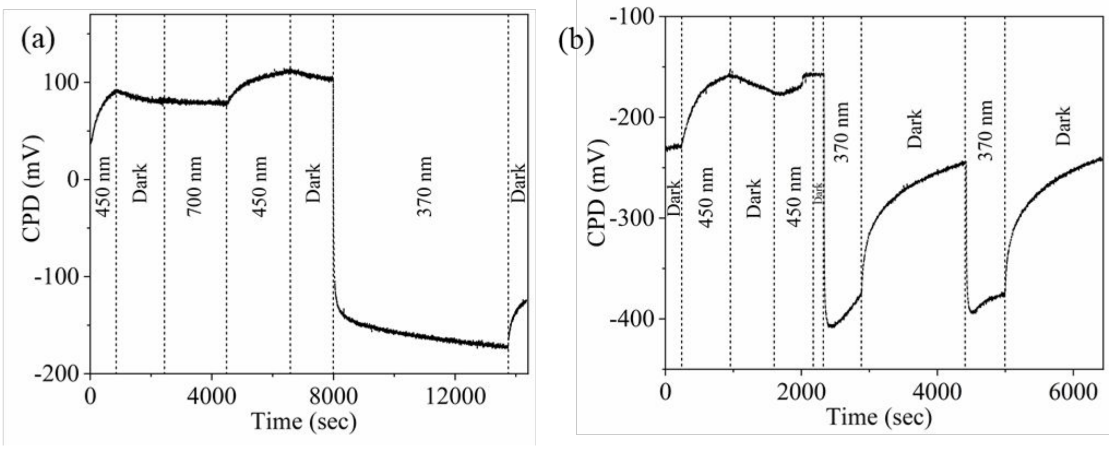

Figure S5. Photo-CPD response of Ag coated with (a) 10 nm and (b) 20 nm of ZnO. Each vertical dotted line designates a transition of the lighting conditions (dark, 370 nm, 450 nm or 700 nm). The CPD changes in opposite directions with response to 450 or 370 nm light, for 700 nm no change in the CPD was received. The transients to stabilization also have different time scales. Measurements were done in a faraday cage with LED lighting in ambient air.
